# Psychological Strain and Suicide Rumination Among University Students: Exploring the Mediating and Moderating Roles of Depression, Resilient Coping, and Perceived Social Support

**DOI:** 10.3390/healthcare13151875

**Published:** 2025-07-31

**Authors:** Nuri Türk, Mustafa Özmen, Sümeyye Derin

**Affiliations:** 1 Department of Guidance and Psychological Counselling, Faculty of Education, Siirt University, 56100 Siirt, Türkiye; nuri.turk@siirt.edu.tr; 2 Department of Child Care and Youth Services, Vocational School of Health Services, Bingöl University, 12000 Bingöl, Türkiye; mozmen@bingol.edu.tr; 3 Youth Studies Research & Application Center, Sakarya University, 54050 Sakarya, Türkiye; 4 Department of Psychology, Faculty of Humanities and Social Sciences, Sakarya University, 54050 Sakarya, Türkiye

**Keywords:** suicide rumination, psychological strain, depression, resilient coping, perceived social support

## Abstract

**Background/Objectives:** Suicide is among the biggest causes of death in the world. In recent years, suicide rates have increased remarkably in developing countries such as Türkiye. Therefore, there is a need to understand the psychological mechanisms underlying suicidal ideation and behaviors. Within this context, this study aimed to examine the complex relationships between psychological strain and suicide rumination. **Methods**: The study was conducted on 470 university students because young adults constitute the largest suicide risk group in Türkiye. **Results**: The findings showed that psychological strain was a significant predictor of suicide rumination. Additionally, depression was found to play a mediating role between psychological strain and suicide rumination. Furthermore, both resilient coping and perceived social support were shown to play a moderating role in the relationships among psychological strain, depression, and suicide rumination. The results confirmed the Strain Theory of Suicide in a sample from Türkiye. **Conclusions**: These findings are expected to contribute to psychologists, psychiatrists and public health specialists’ development of suicide prevention and intervention programs for university students. These suicide prevention and intervention efforts may focus on enhancing resilient coping and perceived social support in combating psychological strain and depression.

## 1. Introduction

Suicide is seen as a common public health problem in many countries around the world, with 726,000 people dying by suicide every year [1]. Suicide attempts are known to be at least 20 times more common than completed suicides [2]. Furthermore, according to a study conducted in 2018, there are at least 135 people who directly and indirectly recognize a person who has died by suicide [3]. Therefore, approximately 100 million people are exposed to the effects of suicide every year. This situation reveals the breadth of the impact of suicide, which is characterized as a social bomb [4].

Suicide is the third leading cause of death, especially among individuals aged 15–29 [1]. Suicide cases among university students in this age group have shown a remarkable increase in the last 10 years [5]. Türkiye is among the countries where such an increase is observed. According to 2023 suicide reports, Türkiye has reached the highest suicide rate (4.94 per 100,000) in its history [6]. Within this rate, the group with the highest number of suicide cases includes individuals between the ages of 20 and 29. These individuals in the quarter-life crisis age range are assumed to be going through a chaotic period due to identity confusion, uncertainty about the future, and conflicts in romantic and family relationships [7]. This transition period, during which it is difficult to respond to the requirements of life, increases the likelihood of experiencing various mental problems that may result in suicide [8]. In a study conducted on young adults, mostly university students in Türkiye, psychological vulnerability and emotional problems (depression, anxiety, somatization) were found to be significant predictors of suicidal ideation [9]. According to another study conducted with Turkish university students, defeat, entrapment, and thwarted belongingness were reported to be significantly positively associated with suicidal ideation [10].

To prevent the suicidal thoughts and behaviors of university students in Türkiye, research is needed to discover new risk and protective factors. Although thousands of studies have been conducted on suicide, new theoretical and practical research continues in light of the debate on the predictability and randomness of suicide [11,12]. Within this context, there are many first- and second-generation theories of suicide used to explain suicide [13]. One of these theories is the Strain Theory of Suicide (STS), developed by Zhang [14]. According to Zhang, psychological strain caused by four different sources leads to suicide [14]. These sources include value strain, aspiration strain, deprivation strain, and coping strain. Value strain refers to the conflict between social values that are of similar importance to the individual, while aspiration strain arises from the conflict between the actual situation and the individual’s aspirations. Deprivation strain is the individual’s feeling that he/she leads a worse life than people who he/she thinks have similar backgrounds, and coping strain is the individual’s feeling of helplessness in the face of life crises [15].

Psychological strain refers to the strain experienced by individuals when confronted with two equally important and contradictory situations. This strain can disrupt the psychological balance of the individual and cause psychological pain accompanied by disappointment, depression, and restlessness. Individuals who want to get rid of psychological pain and regain balance may see suicide as a solution [16]. Significant positive correlations between psychological strain and suicidal ideation have been found in university students from different cultures [17]. Within this context, the present study suggests that psychological strain may predict suicidal rumination, which includes suicidal ideations. When suicidal ideations and plans become repetitive, they turn into suicidal rumination [18]. Suicide-specific rumination is known to be the strongest risk factor for lifelong suicide attempts [19]. Suicide rumination includes mental obsessions during the transition from suicidal ideations to suicidal behaviors. Therefore, studies on suicidal rumination contribute to the identification of high-risk groups in different cultures by determining the frequency and severity of suicidal ideations [20,21].

Depression, which is associated with both rumination and psychological strain [22], is shown to be among the important risk factors for suicide. Meta-analysis studies on mental health risk factors of university students, in particular, show that depression is still effective in explaining suicidal ideation and behavior [23,24]. Additionally, psychological strain is known to be a significant predictor of depression in university students [25]. Studies show that the relationship between psychological strain and suicidal ideation is explained by depression [26]. Additionally, depressive thinking and rumination are often seen together [27,28]. Indeed, Nolen-Hoeksema et al. argue that rumination is a form of repetitive negative thinking in which the individual focuses his/her attention on depressive symptoms [29]. Rumination, a cognitive vulnerability factor, increases the duration and severity of depression and strengthens the possibility of suicide [22]. Furthermore, depression was found to significantly predict suicide rumination according to a study conducted on university students [30]. Consequently, psychological strain predicts depression, while depression predicts suicide rumination. Within this context, it is thought that depression could mediate the relationship between psychological strain and suicide rumination.

Depression plays a mediating role in the relationship between coping strain, which is a sub-dimension of psychological strain, and suicidal ideation [31]. This finding shows the importance of the inadequacy of coping mechanisms in psychological strain leading to depression. Coping methods accompanied by resilience are especially known to be protective factors against suicide in university students [32]. Furthermore, according to an empirical study, resilient coping was found to reduce the risk of suicide [33]. Resilient coping is one of the effective methods for adaptively overcoming strain and stress. Individuals with resilient coping are goal-oriented individuals who believe that they can cope with challenging circumstances [34]. Studies conducted on university students have found that resilient coping has positive correlations with optimism and life satisfaction and negative correlations with depression and anxiety [35,36]. Therefore, resilient coping may moderate the effect of psychological strain on depression by buffering inadequate coping caused by psychological strain.

One of the protective factors against suicide is perceived social support. Perceived social support has a significant negative relationship with suicide as well as with many risky behaviors [37]. Additionally, it is known that social support moderates the effect of psychological strain on suicidality in university students [38]. Therefore, perceived social support may have a moderating effect on depression predicted by psychological strain and suicide rumination. Indeed, perceived social support plays a critical role in coping with depression in university students [39]. University students who can develop healing social relationships have less difficulty coping with depressive moods. This coping method reduces the effect of depression on hopelessness, which is one of the main causes of suicidal thoughts [40]. Within the framework of this literature, perceived social support is expected to play a moderating role in the relationship between depression and suicide rumination.

### The Present Study

Psychological strain, the main concept of the Strain Theory of Suicide (STS), is seen as the source of the pain that leads to suicide. The pain and strain that occur as a result of the conflict between the realities of life and the ideals of individuals can increase the frequency and severity of suicidal ideations [16]. However, the variables underlying this linear relationship between psychological strain and suicide are not yet sufficiently known. Therefore, the present study aimed to examine the role of depression, resilient coping, and perceived social support in the relationship between psychological strain and suicide rumination. Additionally, there is a need for in-depth research on the causes and consequences of suicide among university students, who are among the highest suicide risk age groups in Türkiye. Therefore, this study is expected to guide suicide prevention studies for university students going through a challenging period full of uncertainties.

In this study, the mechanisms underlying the relationship between psychological strain and suicide rumination were examined. Within this context, based on the theoretical and empirical findings in the literature, the following research questions (RQs) were tested in the present study:

1. Does psychological strain predict suicide rumination?
2. Does depression have a mediating effect on the relationship between psychological strain and suicide rumination?
3. Does resilient coping have a moderating effect on the relationship between psychological strain and depression?
4. Does perceived social support have a moderating effect on the relationship between depression and suicide rumination?

## 2. Materials and Methods

### 2.1. Participants

This study was conducted with 470 university students, taking into account the minimum number recommended by G*Power, version 3.1. Of these, 73.6% were female (n = 346) and 26.4% (n = 124) were male. Their ages ranged from 16 to 47 years, with a mean age of 24.93 (SD = 3.77). According to socioeconomic status, 105 (22.3%) participants reported low socioeconomic status, 354 (75.3%) reported medium socioeconomic status, and 11 (2.3%) reported high socioeconomic status.

### 2.2. Instruments

- Sociodemographic data. It was administered to obtain information about the sociodemographic characteristics (e.g., age, gender etc.) of the participants.
- Psychological Strain Scales-Short Form (PSS-SF). PSS-SF was developed by Zhang et al. [41]. The short form of PSS-SF was developed by Huen et al. [42]. PSS-SF was adapted into Turkish by Özmen et al. (43/submitted for publication). Each item is scored between 1 (strongly disagree) and 5 (strongly agree). PSS-SF is a 5-item scale developed to determine the level of psychological strain experienced by the person. High scores indicate high levels of psychological strain. Sample item: ‘Even if I can’t change, I would like to live in a better family.’ PSS-SF has shown good reliability in previous studies (α = 0.80; [42]). The psychometric properties of PSS-SF were examined while it was adapted to Turkish by Özmen et al. [43]. The results showed that, as in the original version, the scale had a single-factor structure and good fit values. Furthermore, the current study re-examined the scale’s CFA and reliability values. The CFA of the scale showed that the fit values were at an excellent level (χ^2^/df = 1.88; RMSEA = 0.04; CFI = 0.99; IFI = 0.99; GFI = 0.99; NFI = 0.98; TLI = 0.98; RFI = 0.96). PSS-SF has shown reliability above the acceptance limits in the current study (α = 0.72).
- Suicide Rumination Scale (SRS). The SRS is a 6-item scale developed to assess recurrent suicidal ideation [18,44]. The SRS is scored from 1 (strongly disagree) to 5 (strongly agree). High scores on the scale indicate high ruminative suicidal ideation. Sample item: “I have difficulty getting suicidal thoughts out of my mind”. In this study, the SRS demonstrated excellent reliability (α = 0.95).
- Depression Anxiety Stress Scale 8 (DASS-8). The DASS-8 [45] comprises eight items and three subscales: depression (e.g., “I feel down and blue”; three items), anxiety (e.g., “I worry about situations where I might panic and make a fool of myself”; three items), and stress (e.g., “I feel under a lot of stress”; two items). The cut-off points for depression and anxiety were normal (0–3), moderate (4–6), and severe (7–9), while the cut-off points for stress were normal (0–2), moderate (3–4), and severe (5–6). This study examined the validity and reliability of the scale.
- Brief Resilient Coping Scale (BRCS). The BRCS [34,46] was used to assess resilient coping and was adapted into Turkish by Özmen et al. (43/submitted for publication). Each item is scored between 1 (does not describe me at all) and 5 (describes me very well). The BRCS is a 4-item scale that assesses a person’s level of resilient coping in the face of stressful and destructive life events. Sample item: ‘I believe that I can develop positively by coping with difficult situations.’ Previous studies have also shown that BRCS has good reliability (α = 0.85; [47]). The psychometric properties of BRCS were examined while it was adapted to Turkish by Özmen et al. [43]. The results showed that, as in the original version, the scale had a single-factor structure and good fit values. Furthermore, the current study re-examined the scale’s CFA and reliability values. The CFA of the scale showed that the fit values were at an excellent level (χ^2^/df = 2.56; RMSEA = 0.06; CFI = 0.99; IFI = 0.99; GFI = 0.99; NFI = 0.99; TLI = 0.99; RFI = 0.98). The scale showed good reliability in the current study (α = 0.84).
- Perceived Social Support Questionnaire (F-SozU K-3). The F-SozU K-3 [48] is a 3-item scale developed as a brief screening instrument. Each item is scored between 1 (not true at all) and 5 (very true). The F-SozU K-3 assesses the level of social support perceived by the individual in the face of any negative experience. Sample item: ‘When I feel down, I know who I can go to without hesitation.’ The F-SozU K-3 showed good reliability in the original study (α = 0.80; [48]). This study examined the validity and reliability of the scale.

### 2.3. Procedure

The first author obtained approval for this study from the Siirt University Ethics Committee (Date: 9 January 2025, reference number: 8241). Data collection took place in February and March 2025. The sample consisted of active students studying in the department of social work. The data were collected via the WhatsApp application in academic classrooms where university students were present. A consent form was presented to all participants both verbally during the application phase and in writing at the beginning of the questionnaire. The questionnaire was administered to university students in a quiet environment free of distracting stimuli. All participants were informed that they could withdraw from the study at any stage. No reward, points, or gifts were given to the participants, as this would not align with normative values in Türkiye. The participants were assured that no personal data would be collected and that the data would be analyzed in groups. All stages of the study were conducted in accordance with the provisions of the Declaration of Helsinki.

### 2.4. Data Analysis

IBM SPSS Version 27 and AMOS Version 25 were used for data analysis. Data analysis consisted of two stages. In the first stage, the DASS-8 and F-SozU K-3 were adapted. Analyses related to the adaptation of the DASS-8 and F-SozU K-3 into Turkish were performed. It was assumed that a model with an RMSEA value <0.05 indicates a good model, while values between 0.05 and 0.08 indicate acceptable fit. Other modification fit index values CFI, RFI, GFI, IFI, NFI, and TLI > 0.95 were accepted as indicators of good fit [49,50,51]. The model was then tested in the second stage. First, the participants (n = 22) who incorrectly marked the control item were excluded from the analysis, as this would reduce the reliability of the data. Second, the normality assumptions of the data were examined [52]. Third, the mediation effect of depression was tested using the SPSS PROCESS macro model 4 proposed by Hayes [53]. Fourth, the SPSS PROCESS macro model 21 proposed by Hayes [53] was used to check whether the conditional indirect effect of perceived social support and resilient coping in the relationship between psychological strain and suicidal rumination was significant. Fifth, for the significance of direct and indirect effects, confidence intervals not covering zero with 5000 resamples were taken as a reference [54].

## 3. Results

### 3.1. Psychometric Properties of DASS-8 and F-SozU K-3

Translation and back-translation procedures were applied during the adaptation of the scales to Turkish [55]. The scale items were translated into Turkish by three field expert academicians fluent in Turkish and English. Two field expert academicians reviewed the translation in terms of clarity and cultural appropriateness of the questions. To examine the grammar of the translated scales, they were back-translated into English by two academicians working in the English department. The suitability of the scales for Turkish was examined by two Turkish teachers. Necessary corrections were made to the scales in line with the feedback provided after all examinations. In the validity and reliability analyses of the scales, Exploratory Factor Analysis (EFA) was performed with 230 data points; following this, Confirmatory Factor Analysis (CFA) was performed with 240 data points. 

According to the EFA results for the DASS-8 scale, the KMO value was 0.89 and the *p* value in the Bartlett test was significant (910.623 (*p* < 0.000). The scale items explained 51% of the total variance. The factor loadings of the scale items ranged between 0.48 and 0.78. CFA of the scale showed that the fit values were above the acceptance limits (χ^2^/df = 2.52; RMSEA = 0.08; CFI = 0.97; IFI = 0.97; GFI = 0.96; NFI = 0.95; TLI = 0.95; RFI = 0.92). To examine convergent validity, the correlation coefficients between the total score of DASS-8 and other research variables were calculated. Psychological distress, which is the total DASS-8 score, is associated with depression (r = 0.87), anxiety (r = 0.90), and stress (r = 0.86). SRS is also moderately correlated with depression (r = 0.48), anxiety (r = 0.38), and stress (r = 0.35). The relationship between DASS-8 and BRCS confirms discriminant validity. BRCS correlates with depression (r = −0.21), anxiety (r = −0.24), and stress (r = −0.17) with low magnitude. Additionally, as a result of the analysis, the CR and AVE values of the scale were found to be 0.93 and 0.57, respectively. The DASS-8 indicated good reliability for depression (α = 0.81), anxiety (α = 0.81), and stress (α = 0.78) in the present study. The total DASS-8 score, indicating psychological distress, also has a good level of reliability (α = 0.89).

According to the EFA results for the F-SozU K-3 scale, the KMO value was 0.62 and the *p* value in the Bartlett test was significant (97.070 (*p* < 0.000). The scale items explained 40% of the total variance. The factor loadings of the scale items varied between 0.52 and 0.79. CFA indicated excellent fit (df = 0.00/0 = 0.00, CFI = 1.00, GFI: 1.00, NFI = 1.00, NFI = 1.00, RMSEA = 0.00, SRMR = 0.00). Similarly, the F-SozU K-3 also had excellent fit index values in the original study [48]. However, since the factor loadings are high as in the original study [48], all three items of the scale can be accepted as meaningful indicators. To examine convergent validity, correlation coefficients between the F-SozU K-3 score and other research variables were calculated. F-SozU K-3 was moderately correlated with BRCS (r = 0.30) and SRS (r = −0.27), which confirms convergent validity. In addition, F-SozU K-3 is associated with depression (r = −0.18), anxiety (r = −0.18), and stress (r = −0.15) in small dimensions. Furthermore, as a result of the analysis, the CR and AVE values of the scale were found to be 0.82 and 0.60, respectively. The F-SozU K-3 showed good reliability in the present study (α = 0.82). As a result, it can be said that the validity and reliability values of the F-SozU K-3 scale are at an acceptable level.

### 3.2. Model Analyses

Before testing model 21, we examined the mean, standard deviation, normality values, and correlation coefficients of the variables. Table 1 shows that the research variables met the normality assumptions. However, psychological strain was negatively associated with perceived social support and resilient coping, whereas it was positively associated with depression and suicide rumination. Perceived social support is negatively related to depression and suicide rumination, and positively related to resilient coping. Finally, depression is positively related to suicide rumination and negatively related to resilient coping.

### 3.3. Testing for Mediation Effect

**Figure 1:**
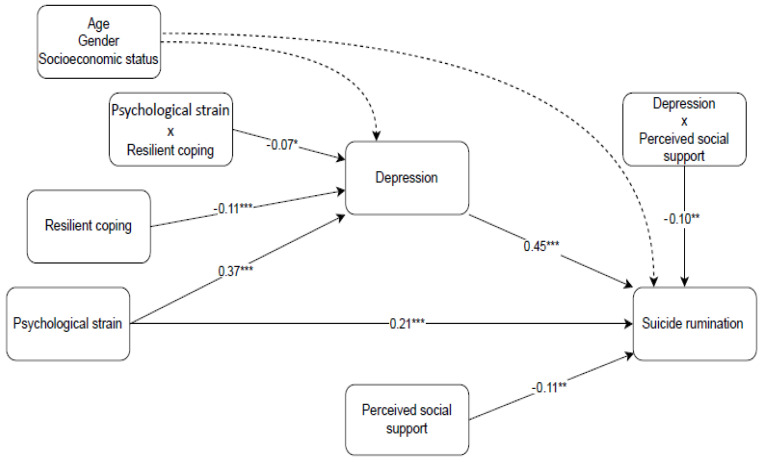
The moderated mediation model. Path coefficients are standardized regression coefficients. * *p* < 0.10, ** *p* < 0.01, *** *p* < 0.001.

For RQ1, the total effect was analyzed to examine whether psychological strain predicts suicidal rumination. After controlling for age, gender, and socioeconomic status, psychological strain strongly positively predicted suicide rumination in total (*β* = 0.44, t = 8.87, *p* < 0.001). For RQ2, whether depression has a mediating effect on the relationship between psychological strain and suicidal rumination was tested. SPSS macro model 4 was used to test this model (Figure 1). First, the predictive relationships in the variables were discussed, and then the mediating effect was analyzed. After controlling for age, gender, and socioeconomic status, psychological strain positively predicted depression (*β* = 0.39, *t* = 10.33, *p* < 0.001). Similarly, psychological strain positively predicted suicide rumination (*β* = 0.25, *t* = 4.90, *p* < 0.001). Depression positively predicted suicide rumination (*β* = 0.47, *t* = 8.54, *p* < 0.001). The total effect between psychological strain and suicidal rumination was also positively significant (*β* = 0.44, *t* = 8.87, *p* < 0.001). Finally, after controlling for covariates, depression had a strong mediating effect on the relationship between psychological strain and suicidal rumination (indirect effect = 0.19, *SE* = 0.04, 95% CI = [0.1201, 0.2608]).

### 3.4. Moderated Mediation Analyses

**Figure 2:**
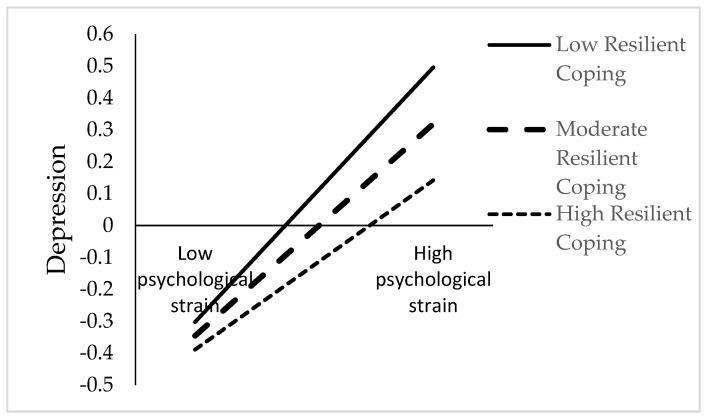
Interaction between psychological strain and resilient coping on depression.

PROCESS macro model 21 was used to conduct moderated mediation analyses. For RQ3, whether resilient coping moderated the first stage of the mediator in the relationship between psychological strain and suicidal rumination was tested. As seen in Table 2 and Figure 2, there is a moderating effect of resilient coping on the effect of psychological strain on depression. The association of psychological strain with depression was stronger among individuals with low levels of resilient coping (*β* = 0.44, *SE* = 0.05, 95% CI = [0.3438, 0.5356]) than among individuals with high levels of resilient coping (*β* = 0.29, *SE* = 0.05, 95% CI = [0.1912, 0.3938]). However, the indirect association of psychological strain via depression with suicide rumination was stronger among individuals with low levels of resilient coping (*β* = 0.21, *SE* = 0.04, 95% CI = [0.1346, 0.2855]) than among individuals with high levels of resilient coping (*β* = 0.14, *SE* = 0.03, 95% CI = [0.0769, 0.2068]).

**Figure 3:**
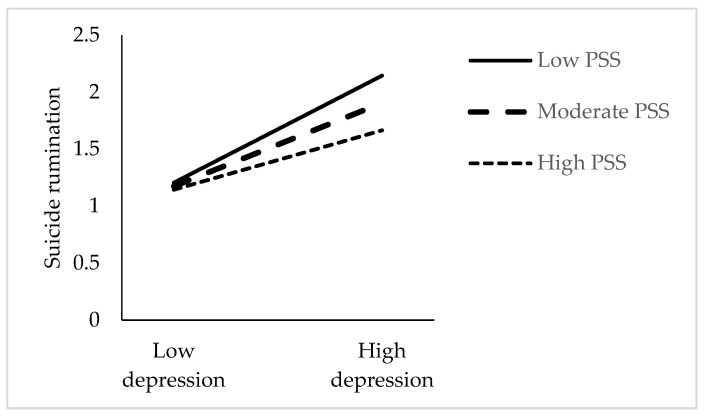
Interaction between depression and perceived social support (PSS) on suicide rumination.

For RQ4, whether PSS moderates the second stage of the mediator in the relationship between psychological strain and suicidal rumination was examined. As seen in Table 2 and Figure 3, there is a moderating effect of PSS on the effect of depression on suicidal rumination. The association of depression with suicidal rumination was stronger among individuals with low levels of PSS (*β* = 0.57, *SE* = 0.07, 95% CI = [0.4439, 0.7078]) than among individuals with high levels of PSS (*β* = 0.32, *SE* = 0.07, 95% CI = [0.1786, 0.4603]). However, the indirect association of psychological strain via depression with suicide rumination was stronger among individuals with low levels of PSS (*β* = 0.23, *SE* = 0.05, 95% CI = [0.1286, 0.3398]) than among individuals with high levels of PSS (*β* = 0.13, *SE* = 0.03, 95% CI = [0.0625, 0.1990]).

## 4. Discussion

This study examined the complex relationships between psychological strain and suicide rumination. Previous studies have shown that suicidal ideation [56,57], suicidal intent [58], and suicidal behavior [59] are associated with psychological strain. However, no previous studies have been found that examine the relationship between psychological strain and suicide rumination and the possible psychological variables that may affect this relationship. Considering this gap in the literature, the present study aims to examine the mediating role of depression and the moderating role of resilient coping and perceived social support in the relationship between psychological strain and suicide rumination. The results confirmed the direct and indirect effects of psychological strain on suicide rumination. The findings showed that depression mediated the relationship between psychological strain and suicide rumination, and resilient coping and perceived social support had a moderating role in this indirect relationship. In addition, the present study showed that the Strain Theory of Suicide can be used to explain suicide rumination in Turkish youth who continue their university education.

The psychometric properties of the DASS-8 and F-SozU K-3 scales were tested in the Turkish population. The EFA results for the construct validity of the DASS-8 scale showed that the scale comprises a three-factor structure in the Turkish population, similar to the results of previous studies. According to the EFA results for the F-SozU K-3, the scale consists of a single dimension, which is consistent with the previous study [48]. Then, a separate CFA was applied for both scales, and it was determined that the fit index values of DASS-8 were above the acceptance limits, while the F-SozU K-3 had excellent fit index values [49,50,51]. The goodness of fit values of both scales are similar to previous studies [45,48,60]. In the current study, the convergent and discriminant validity of the scales were also examined. The CR and AVE values [61,62] of DASS-8, the significant relationship between the DASS-8 total score and SRS and depression, anxiety, and stress confirm the convergent validity of the scale. Similarly, the CR and AVE values [61,62] of the F-SozU K-3 and the significant relationship between the scale and BRCS and SRS scores confirm the convergent validity. In addition, the significant correlation of both scales with depression, anxiety, and stress, which is less than 0.85, confirms the discriminant validity of the scale [50]. The internal consistency coefficients of both scales are consistent with the results of previous studies [48,60], and the results show that the scales are reliable. In summary, the results show that the DASS-8 and F-SozU K-3 are valid and reliable measurement tools in the Turkish university student population.

Whether psychological strain predicts suicide rumination in RQ1 has been tested. The findings showed that psychological strain significantly predicted suicide rumination in a positive direction. This finding indicates that Turkish university students who experience psychological strain may have difficulty in reaching a solution, and repetitive thoughts about ending their lives may increase. This finding is consistent with the results of previous studies [22,56,57]. There are also studies in the literature reporting that rumination increases in individuals who experience stressful life events [63]. Psychological strain involves both internal conflicts and strain that require resolution, as well as inadequate coping mechanisms [16]. Furthermore, university students are a population that has to deal with many developmental tasks [64], challenging peer relationships [65], and academic [66] and financial stress [65,67]. Intense strain felt in the face of different problem areas and inadequate coping mechanisms can hinder students’ ability to make healthy cognitive assessments and produce effective solutions. Individuals who are unable to achieve internal balance may feel trapped and develop mental obsessions with repetitive suicidal thoughts (suicide rumination), considering suicide as a solution. However, this finding was obtained with the total score of psychological strain. Some university students may not experience psychological strain in all four areas, and the areas where they do experience psychological strain may vary. Therefore, a future, more detailed investigation of the effects of psychological strain sources on suicide rumination will contribute to the expansion of knowledge on this subject.

For RQ2, whether depression would mediate the relationship between psychological strain and suicide rumination was tested. As expected, the findings showed that psychological strain has an indirect effect on suicide rumination through depression. In this case, it can be said that depression explains psychological strain leading to suicide rumination among Turkish university students. This finding is in accordance with studies reporting that depression increases as the level of psychological strain increases [68,69] and that individuals with depression have more suicidal thoughts [70]. Psychological strain may be accompanied by feelings such as frustration, hopelessness, and helplessness [16]. Challenging emotions experienced with psychological strain and decreased emotional resilience [69] may trigger depression. Depression may lead to increased suicide rumination because it reduces positive emotions [71], increases rumination, and reduces the use of functional and flexible emotion regulation strategies [72]. In addition, the current research finding supports previous research results showing that depression mediates the association between psychological strain and suicidal ideation in various populations [22,57]. Negative emotions that arise with psychological strain may deepen with depression. In the face of intense, challenging emotions, an individual’s emotion regulation mechanism may become dysfunctional. This situation may negatively affect the well-being of university students and increase the tendency of repetitive thinking about suicide. Although the current study provides explanatory information about the role of depression in the relationship between psychological strain and suicide rumination, this relationship was examined in a one-way manner because a cross-sectional design was used in the study. These variables have a dynamic structure and interact with each other. Future research using a longitudinal design may provide deeper insights into the relationship between variables and change over time.

Findings showed that resilient coping moderated the relationship between psychological strain and depression. This finding supports recent research reporting significant negative associations between resilient coping and depression [73,74]. In addition, it is reported in the literature that those with high levels of resilient coping experience less psychological strain [75]; indeed, one of the four sources of psychological strain is inadequate coping [16]. Therefore, individuals with high resilient coping skills may experience psychological strain in fewer areas. In addition, when these individuals experience psychological strain, they can make active efforts to make progress by using their own resources and resort to new ways for solutions [46]. Making an effort and the search for new solutions, which develop due to resilient coping, can make individuals feel more hopeful [76]. Individuals with high levels of hope experience fewer depressive feelings and thoughts [77]. In conclusion, these relationships support the buffering role of resilient coping in the effect of psychological strain on depression. For this reason, intervention programs could be developed that aim to develop resilient coping skills, especially in university students experiencing psychological strain.

Finally, this study showed that perceived social support has a moderating effect on the relationship between depression and suicide rumination. In other words, higher levels of perceived social support are associated with a reduced effect of depression on suicide rumination. This finding supports the results of studies reporting a negative significant relationship between depression and perceived social support [78] and demonstrating the protective function of perceived social support towards suicidal ideation [79]. When individuals with depressive symptoms have a high perception of social support, they can cognitively reappraise events [80,81] and use more flexible coping strategies [75]. This situation may reduce the level of depression by increasing the use of individuals’ personal resources. A decrease in depression levels is characterized by positive cognitive and emotional changes, which may also reduce suicide rumination. In addition, social support contributes to the individual’s sense of belonging to his/her wider environment, society, and the world—from his/her family to the world. Social support has an especially important place in Turkey, where collectivist cultural values are dominant [82]. Therefore, social support may decrease suicide rumination by increasing belongingness and decreasing feelings of loneliness in depressed Turkish university students. However, this finding does not provide detailed information about which sources the participants received social support from and to meet which needs [83]. Future studies may contribute to the expansion of the literature on this subject by addressing these dimensions of the social support source.

The findings of the present study should be evaluated while considering its limitations. The first limitation of the study is that causal inferences cannot be made using the findings due to the study’s cross-sectional design. Experimental and longitudinal studies can be planned in the future to overcome this limitation. The second limitation is that the study sample consists of university students in the Southeast region of Turkey. Therefore, the results of the study cannot be generalized to all university students or different age groups across Turkey. In the future, studies with participants from different regions and age groups [84] can be conducted, and the results can be analyzed comparatively. The third limitation of the study is that the proportion of female participants is higher than that of male participants. The convenience sampling method was used, and the volunteering of the participants was taken into account. Since females volunteered to participate in the study more than males in the accessible population, a sufficient balance could not be achieved in the female–male ratio. Similar to the current study, the inadequacy of male and female representation in different disciplines and research areas is a topic of discussion [85,86]. To address this point, researchers can examine the factors affecting volunteering in psychological research from a gender perspective. Another limitation is that the participants could not respond to the measurement tools under similar conditions because the research data were collected through an online platform. This situation may have increased the effect of external factors and affected participant responses. Finally, the data for this study were collected using self-report instruments. Therefore, the participants may have answered the scales in the direction of social desirability. This limitation can be overcome by using qualitative data collection methods such as observations and interviews in future studies.

## 5. Conclusions

The present study provides important findings that can be used to inform interventions aimed at preventing suicide among university students. In particular, mental health professionals, university administration units, and academicians can ensure that the results of this research are translated into practice through various planned studies. Considering that suicide rumination is one of the determining factors of lifelong suicide attempts [19], identifying university students experiencing psychological strain may be the first step of suicide prevention interventions that can be carried out at universities. In the next step, effective intervention programs can be implemented for students experiencing psychological strain. Preparing the content for these programs, approaches, and techniques can be used to reduce depression, strengthen resilient coping, and increase social support resources instead of focusing only on psychological strain. In addition, mental health professionals could use group therapy while providing services to university students who are experiencing psychological strain and undergoing depressive processes. In addition to reducing psychological strain and depression levels in group therapy processes, students may find the opportunity to gain resilient coping skills and get rid of their feelings of loneliness. Additionally, university administration units could aim to increase perceived social support by designing spaces and planning activities that will increase students’ interactions with their peers and professors. Academics, on the other hand, could plan activities that can be done together with groups in classes and structure courses to strengthen interpersonal bonds. Furthermore, policymakers can act by taking into account the results of this study when determining public health policies. In this way, suicide can be prevented by increasing strategies for coping with psychological strain.

## Figures and Tables

**Table 1:** Descriptive statistics.

	Descriptive Statistics and Reliabilities					Correlations				
Variables	*M*	*SD*	Skewness	Kurtosis	α	1	2	3	4	5
1. Psychological strain	2.76	0.91	0.24	−0.40	0.72	-				
2. PSS	3.67	1.19	−0.70	−0.51	0.82	−0.29 **	-			
3. Depression	1.38	0.81	0.46	−0.67	0.81	0.45 **	−0.18 **	-		
4. Resilient coping	3.67	1.01	−0.66	0.11	0.84	−0.20 **	0.30 **	−0.23 **	-	
5. Suicide rumination	1.56	1.01	2.11	3.66	0.95	0.39 **	−0.27 **	0.47 **	0.31 **	-

Note. PSS = Perceived Social Support; ** *p* < 0.01.

**Table 2:** Conditional effect coefficients predicting suicide rumination and depression.

Variables		*β*	*SE*	t
Mediator—depression				
Predictor: psychological strain		0.37	0.04	9.44 ***
Moderator: resilient coping		−0.11	0.03	−3.22 ***
Interaction: psychological strain x resilient coping		−0.07	0.03	−2.31 *
Outcome—suicide rumination				
Predictor: psychological strain		0.21	0.05	4.07 ***
Mediator: depression		0.45	0.05	8.28 ***
Moderator: perceived social support		−0.11	0.03	−3.25 **
Interaction: depression x perceived social support		−0.10	0.04	−2.94 **
*R* ^2^		0.31		
Different resilient coping values		*β*	*SE*	95% CI
	−1 *SD*	0.44	0.05	[0.344, 0.536]
	Mean	0.37	0.04	[0.290, 0.542]
	+1 *SD*	0.29	0.05	[0.191, 0.394]
Different perceived social support values		*β*	*SE*	95% CI
	−1 *SD*	0.57	0.07	[0.444, 0.708]
	Mean	0.45	0.05	[0.341, 0.554]
	+1 *SD*	0.32	0.07	[0.179, 0.460]

Note. CI = Confidence Interval; SE = Standard Error; * *p* < 0.05; ** *p* < 0.01; *** *p* < 0.001.

## Data Availability

The data supporting this study’s findings are available from the corresponding author upon reasonable request. The data were anonymized, ensuring that there was no breach of privacy. They will be shared in a manner that respects ethical protocols and data protection regulations. The dataset will be accessible only for academic purposes, and any use of the data will recognize the original study and maintain the confidentiality of the participants.

## Abbreviations

The following abbreviations are used in this manuscript:

WHO	World Health Organization
RQ	Research Questions
STS	Strain Theory of Suicide
PSS-SF	Psychological Strain Scales-Short Form
SRS	Suicide Rumination Scale
DASS-8	Depression Anxiety Stress Scale 8
BRCS	Brief Resilient Coping Scale
F-SozU K-3	Perceived Social Support Questionnaire
PSS	Perceived social support
CI	Confidence interval
